# Supplementary material for: Assessment of Therapeutic Potential of a Dual AAV Approach for Duchenne Muscular Dystrophy
Source: Int J Mol Sci. 2023 Jul 13;24(14):11421. doi: 10.3390/ijms241411421 (PMC10380683; doi:10.3390/ijms241411421)
Supplement: Supplementary file 1 [file ijms-24-11421-s001.zip › ijms-2461236-supplementary.pdf]

Figure S1

A

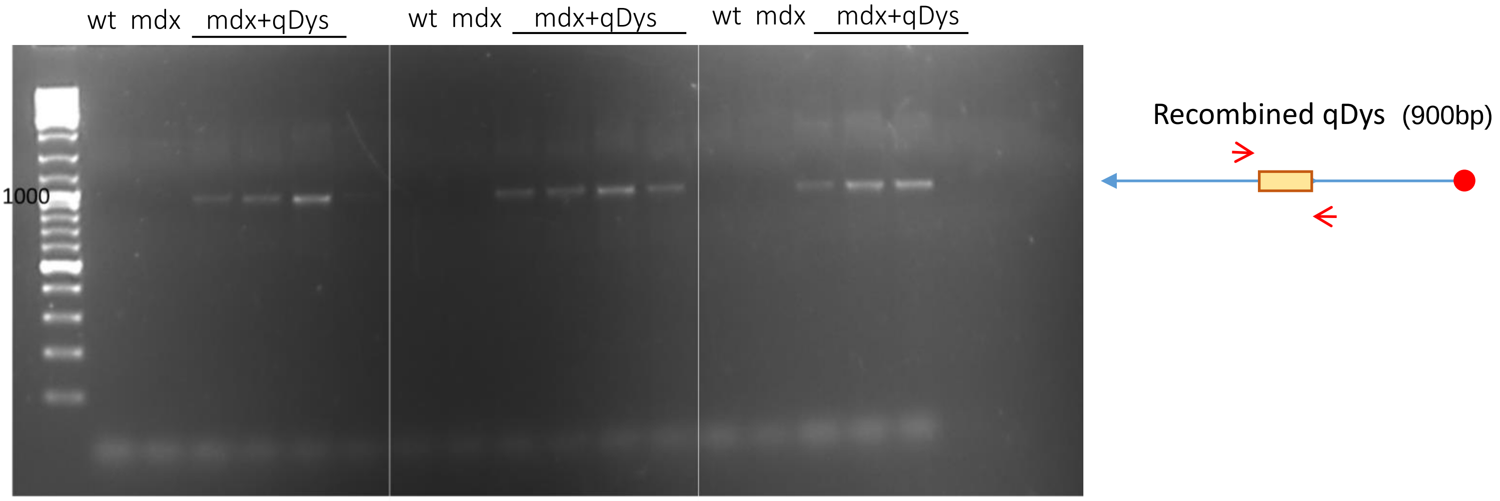

B

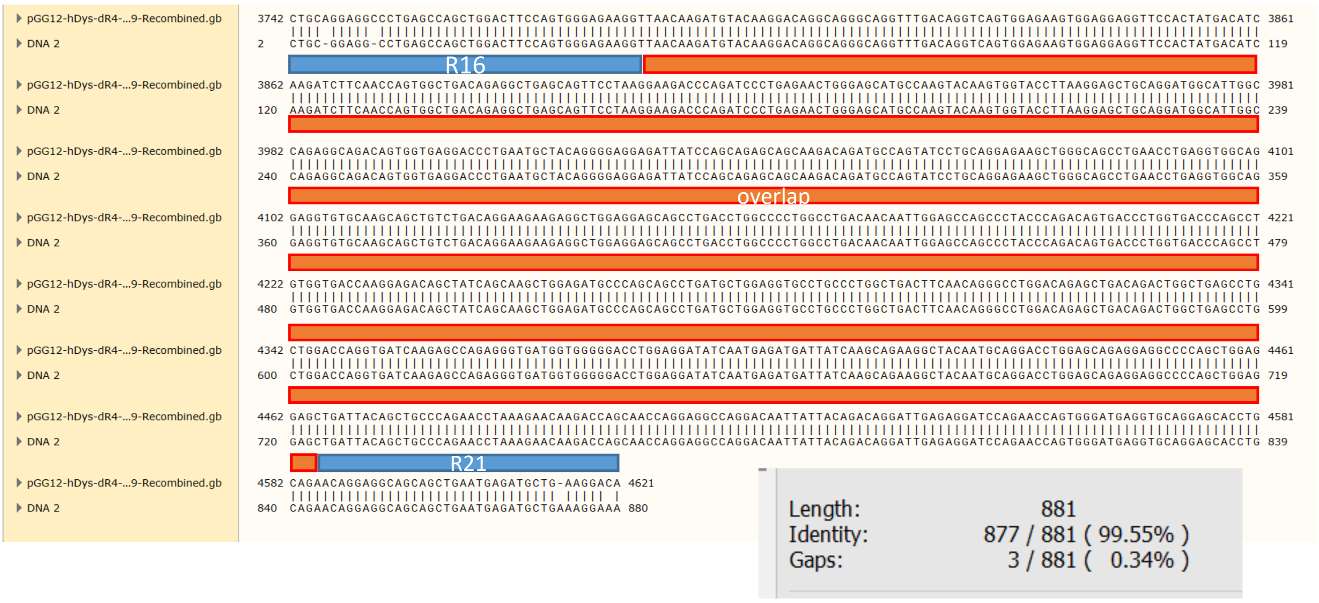

Figure S1.Verification of identity and specificity of the ddPCR amplified recombined product. **A**) SmaI-digested ddPCR amplified product using primers indicated. The 900 bp amplicon was recovered from droplet reaction and loaded onto electrophoretic gel to check the size. **B**) Sequencing of the amplicon purified from gel. The box shows identity % with the recombined expected sequence.

Figure S2

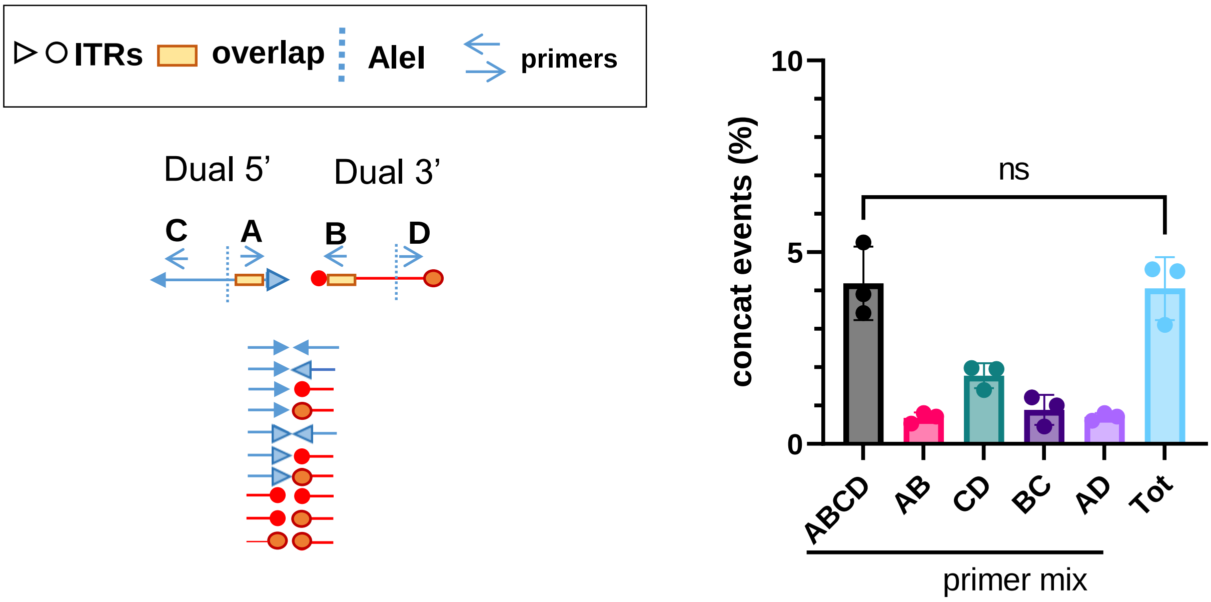

Figure S2. Primer mix optimization for the detection of all concatemerization events.
Mixing all the primers in the same reaction (ABCD) gives the same result than adding up the concatemerization events (Tot) derived from the single reactions containing single combination of primers (AB, CD, BC,AD). Therefore, the mix of all primers can provide an accurate results in one reaction.

Figure S3

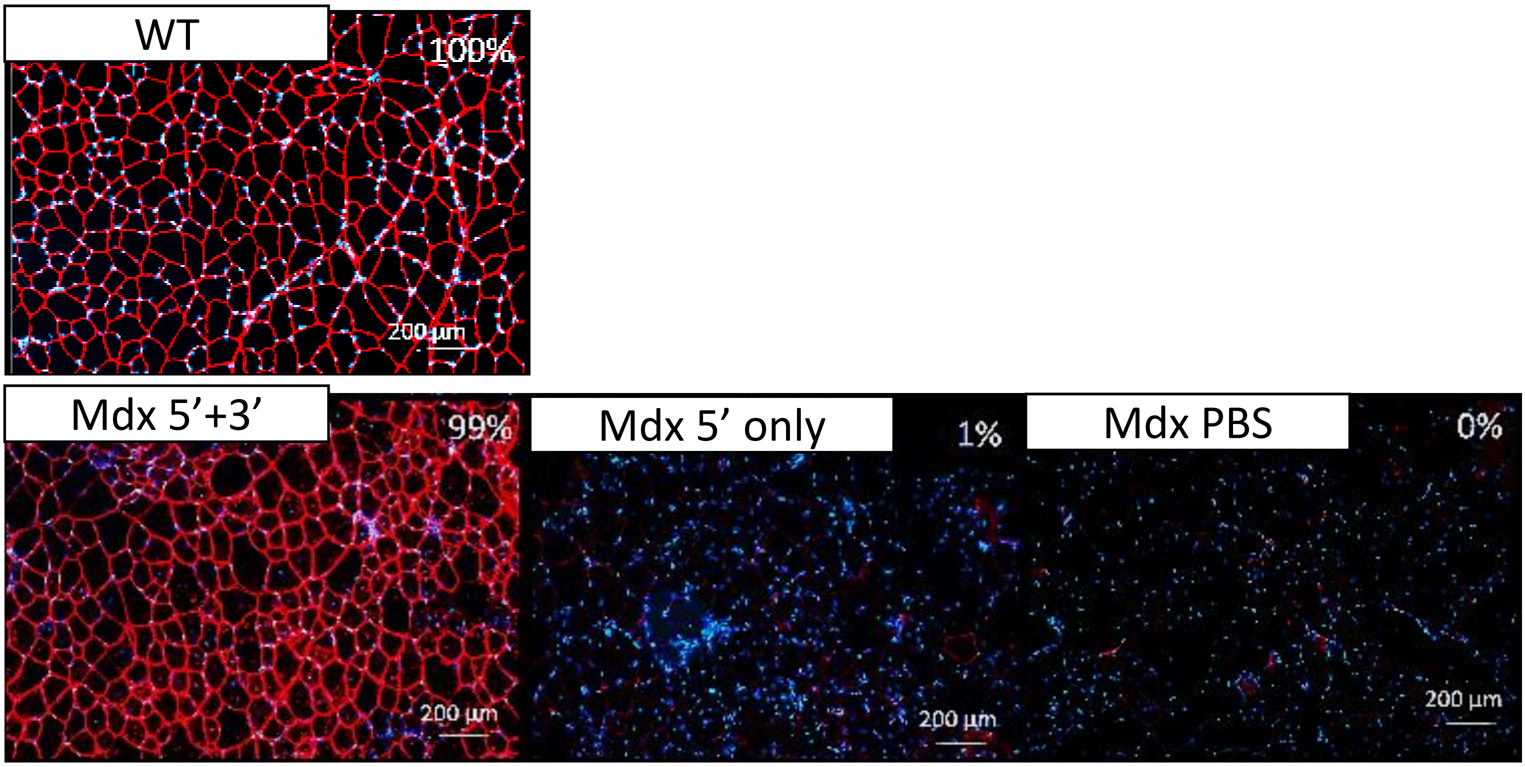

Figure S3. Lack of Dystrophin expression in single vector-injected mdx mice. Representative images of sections of tibialis anterior of DBA2 WT and mdx mice after systemic injection of 5' vector alone or in combination with 3'. Indicated in each picture are % of dystrophin positive fibers.

Table S1. Primers used in ddPCR multiplex analysis.

Target	Fw primer	Rev primer	Probe
**5’**	CAGGAGGCCAGGACAATTATTAC	CCATGCTACTTATCTACGTAAAGC	**2X FAM** TGAGAGGATCCAGAACCAGTGGGA
**3’**	AACCCGCCATGCTACTTATC	TGGAACCTCCTCCACTTCT	**VIC** AAGATGTACAAGGACAGGCAGGGC
**REC**	GCCCTGCAGTCTGCTAC	TCCTTCAGCATCTCATTCAGC	**VIC** TGAGAGGATCCAGAACCAGTGGGA
**TTN**	TTCAGTCATGCTGCTAGCGC	AAAACGAGCAGTGACGTGAGC	**FAM** TGCACGGAAGCGTCTCGTCTCAGTC

Mix1 (VCN single vectors)= 5’+3’+TTN

Mix2 (VCN reconstituted vector)= REC+TTN

Table S2. Primers used for concatemerization analysis.

**A**	CAGGATTGAGAGGATCCAGAAC
**B**	GTTTGACAGGTCAGTGGAGAA
**C**	CAGGTGTTGGCGCTCTAA
**D**	TGAGTTTGGACAAACCACAAC
